# Antioxidant and Anti-Melanogenesis Effects of *Teucrium chamaedrys* L. Cell Suspension Extract and Its Main Phenylethanoid Glycoside in B16-F10 Cells

**DOI:** 10.3390/plants13060808

**Published:** 2024-03-12

**Authors:** Letizia Pruccoli, Benedetta Nicolini, Mariacaterina Lianza, Gabriella Teti, Mirella Falconi, Andrea Tarozzi, Fabiana Antognoni

**Affiliations:** 1 Department for Life Quality Studies, University of Bologna, 47921 Rimini, Italy; letizia.pruccoli2@unibo.it (L.P.); benedetta.nicolini1981@gmail.com (B.N.); mariacaterina.lianz3@unibo.it (M.L.); fabiana.antognoni@unibo.it (F.A.); 2 Department of Biomedical and Neuromotor Sciences, University di Bologna, 40126 Bologna, Italy; gabriella.teti2@unibo.it; 3 Department of Medical and Surgical Sciences, University di Bologna, 40126 Bologna, Italy; mirella.falconi@unibo.it; 4 Biostructures and Biosystems National Institute (INBB), 00136 Rome, Italy

**Keywords:** antioxidant activity, anti-melanogenic activity, B16-F10 cells, cell culture extract, germander, phenylethanoid glycosides, teucrioside

## Abstract

*Teucrium chamaedrys* L. is a typical European–Mediterranean species of the genus Teucrium. Among the phenolic compounds belonging to phenylethanoid glycosides (PGs), teucrioside (TS) is only found in this species, and it was previously demonstrated to be produced by in vitro-elicited cell cultures at levels higher than those found in leaves. However, *T. chamaedrys* cell suspension extracts (Cell-Ex) and pure TS have not been investigated yet for any biological effects. In this study, we evaluated the antioxidant and anti-melanogenesis activity of both Cell-Ex and TS in B16-F10 mouse melanoma cells. The results showed that Cell-Ex inhibited the reactive oxygen species formation evoked in B16-F10 cells by *tert*-butyl hydroperoxide and 5 J/cm^2^ of UVA, as well as the melanin increase stimulated by α-MSH or 20 J/cm^2^ of UVA. In parallel, a TS concentration equivalent to that present in Cell-Ex recorded the same biological effect profile, suggesting the main contribution of TS to the antioxidant and anti-melanogenic properties of Cell-Ex. Both Cell-Ex and TS also modulated the melanogenesis pathway through their ability to inhibit the tyrosinase activity both in a cell-free system and in B16-F10 cells stimulated by α-MSH. These results support the potential cosmeceutical use of Cell-Ex for protection against photooxidative damage and hyperpigmentation.

## 1. Introduction

*Teucrium chamaedrys* L., commonly called germander, is a typical European–Mediterranean species of the genus *Teucrium* [1]. The aerial parts of this species have been widely used in folk medicine for their bitter, astringent, digestive, diuretic, anti-oxidant, anti-inflammatory, and anti-rheumatic properties [2]. Several secondary metabolites such as monoterpenes and diterpenes, saponins, glycosides (iridoids and phenylethanoids), and flavonoids found in *T. chamaedrys* contribute to a wide spectrum of biological and pharmacological effects [1]. Sadly, among these metabolites, neo-clerodane diterpenoids, including teucrin A and teuchamaedryn A, are causes of hepatotoxicity [3]. In this regard, French and Italian authorities have introduced several limitations for use of preparations obtained from *T. chamaedrys* [4].

**Figure 1:**
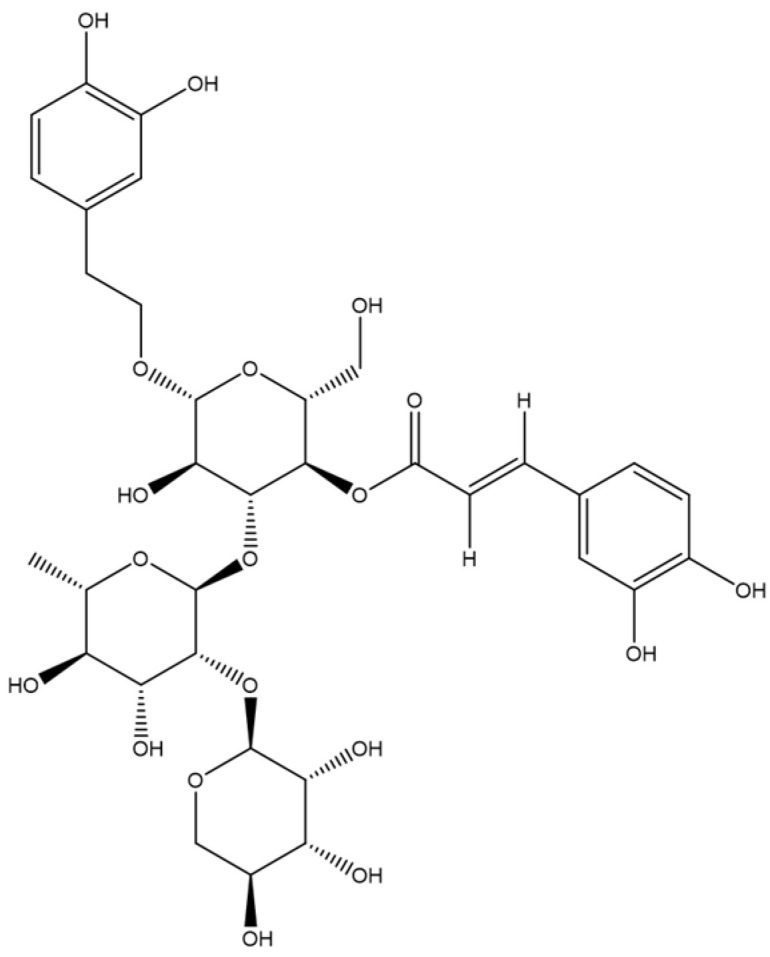
Chemical structure of TS.

The main beneficial effects of *T. chamaedrys* can be attributed to its high concentration of phenylethanoid glycosides (PGs) [1]. Among the PGs, teucrioside (TS, Figure 1) is a new L-lyxose-containing PG found only in this species of *Teucrium* [5,6,7]. Recent studies have shown that extracts from both *T. chamaedrys* leaf and cell cultures have anti-oxidant activity in cell-free systems, indicating the potential benefits of this plant [8,9]. Based on this evidence, there is a growing interest in improving the extraction of antioxidant PGs, avoiding the presence of hepatotoxic neo-clerodane diterpenoids by either bio-guided fractioning strategies or an alternative approach to whole plants [4,9]. Among the relevant approaches, *T. chamaedrys* cell cultures with elicitors such as methyl jasmonate, proline, hydroxyproline, and fungal elicitors have previously been reported to be able to produce PGs at TS levels equivalent to or higher than those found in T. chamaedrys leaf, preventing the formation of hepatotoxic diterpenoids [9].

The peculiar phytochemical profile of *T. chamaedrys* cells prompted us to evaluate their potential as a cosmeceutical. Several in vitro and in vivo studies exhibited a wide array of biological activities, including the antibacterial, antitumor, antiviral, anti-inflammatory, neuroprotective, antioxidant, hepatoprotective, and immunomodulatory ones [10,11]. A tyrosinase inhibitory activity was also demonstrated for this class of metabolites, even though the cosmeceutical properties of in vitro-cultured cells from *T. chamaedrys* and single TS have yet to be studied. Therefore, the aim of this study was to initially evaluate the antioxidant activity of methanolic extracts obtained from a *T. chamaedrys* leaf (Leaf-Ex) and *T. chamaedrys* cell suspension (Cell-Ex), as well as the pure TS isolated from leaves, in B16-F10 mouse melanoma cells. Then, the anti-melanogenesis activity of Cell-Ex and TS was evaluated in the same cellular model in terms of their ability to reduce melanin levels and cellular tyrosinase activity. To achieve oxidative stress in B16-F10 cells, we used *tert*-butyl hydroperoxide (t-BOOH), a lipophilic hydroperoxide, and ultraviolet A (UVA) radiation, given their capacity to generate reactive oxygen species (ROS) as a consequence of the lipid peroxidation occurring in biological membranes. Among shortwave (320–340) and longwave (340–400) UVA, we used longwave UVA, which represents approximately 75% of solar UVA radiation and UVA emitted by tanning lamps [12].

## 2. Results and Discussion

### 2.1. Antioxidant Activity of Leaf-Ex, Cell-Ex and TS

To evaluate the antioxidant effects of the extracts Leaf-Ex and Cell-Ex in B16-F10 cells, we first established the extract concentrations not associated with cytotoxicity using an MTT assay. The treatment of the B16-F10 cells for 24 h with a range of concentrations 1–100 µg/mL did not affect cell viability. Prolonged treatment of the B16-F10 cells for 72 h with both Leaf-Ex and Cell-Ex showed a significant reduction in cell viability at concentrations higher than 30 µg/mL. Thus, a concentration of 10 µg/mL was chosen for the subsequent experiments.

**Figure 2:**
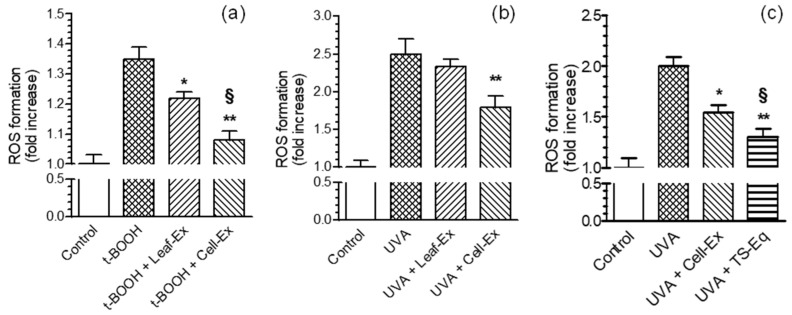
Effects of Leaf-Ex, Cell-Ex and TS-Eq on ROS formation induced by t-BOOH and UVA in B16-F10 cells. Cells were treated with 10 µg/mL of the studied extracts or TS-Eq and 100 µM of t-BOOH (**a**) for 30 min or 5 J/cm^2^ of UVA (**b**,**c**). At the end of the treatment, ROS formation was determined using the fluorescent probe DCFH-DA, as reported in the Materials and Methods section. The ROS formation values are expressed as fold increases with respect to untreated cells. The values are shown as the mean ± SD of three independent experiments (* *p* < 0.05 and ** *p* < 0.01 vs. cells treated with t-BOOH or UVA, § *p* < 0.05 vs. cells treated with UVA and Leaf-Ex or Cell-Ex via a one-way ANOVA with the Bonferroni post hoc test).

The antioxidant activity of the extracts was evaluated against the ROS formation induced by t-BOOH and longwave UVA by fluorescent probe DCFH-DA. Treating the B16-F10 cells with 10 µg/mL of either Leaf-Ex or Cell-Ex significantly decreased the ROS formation induced by t-BOOH for 30 min (Figure 2a). The antioxidant activity of Cell-Ex was significantly higher than Leaf-Ex (inhibition percentage, 77 vs. 37). The concentration of 10 µg/mL of Cell-Ex, but not Leaf-Ex, also showed the ability to inhibit the ROS formation evoked in B16-F10 cells by UVA exposure (Figure 2b).

Regarding these findings, the higher levels of TS found in Cell-Ex (113.5 µg TS/mg Cell-Ex) compared to Leaf-Ex (8.3 µg TS/mg Leaf-Ex) by HPLC-DAD could explain the difference in antioxidant activity observed in B16-F10 cells. Further, the significant antioxidant activity of Cell-Ex during oxidative stress suggests a free radical scavenger action of the phytochemical composition mainly at the membrane level. A recent study evaluated the antioxidant activity of iridoid glycosides and other PGs from the plant *T. chamaedrys* in cell-free systems, recording the strong ability of PGs to inhibit lipid peroxidation using the thiobarbituric acid reactive substance assay [8]. Interestingly, PG verbascoside, which has a chemical structure very similar to TS, demonstrated similar antioxidant activity on lipid peroxidation in immobilized rabbits [13]. Regarding the antioxidant activity of Cell-Ex against longwave UVA, it is also relevant that Cell-Ex shows some absorption for shortwave UVA (absorption peak 327 nm, Figure S1) but not for longwave UVA, highlighting that this antioxidant activity is independent of UVA filter effects. Leaf-Ex and TS have a similar absorption spectrum with a 326 and 329 nm peak, respectively (Figures S2 and S3).

To evaluate the contribution of TS to the antioxidant activity of Cell-Ex against ROS formation induced by UVA, a TS concentration equivalent (TS-Eq) of 1.13 µg/mL to that present in 10 µg/mL of Cell-Ex was used under similar experimental conditions. Remarkably, the ability of TS-Eq to inhibit ROS formation was significantly higher than Cell-Ex (inhibition percentage, 70 vs. 46), suggesting its main contribution to the antioxidant properties of Cell-Ex (Figure 2c). Interestingly, these results also highlight that TS enucleated from Cell-Ex, showing a higher antioxidant activity, probably due to a greater bioavailability in B16-F10 cells.

### 2.2. Anti-Melanogenesis Activity of Cell-Ex and TS

UV radiation and pollutants can damage skin and accelerate skin aging. UVA exposure is an important cause of increased melanogenesis by inducing oxidative stress and damaging the antioxidant defense mechanism in melanocytes [14,15]. Few studies have assessed the ability of PGs to modulate melanogenesis processes. Recently, PGs from Ginkgo biloba leaves have been found to inhibit mushroom tyrosinase activity, highlighting the potential properties of PGs to modulate the melanogenesis processes [16]. It is known that tyrosinase is a rate-limiting enzyme in the melanogenesis pathway. An interesting study also reports that the PG verbascoside did not directly inhibit the activity of mushroom tyrosinase, but it was able to reduce both tyrosinase activity and melanin production in B16 melanoma cells stimulated by α-MSH through a different mechanism [17]. In another study, a cell extract of *Ajuga reptans* L., a species producing a relevant amount of PG, and the pure compounds teupolioside and isoteupolioside were reported to inhibit tyrosinase activity in a cell-free system [18].

**Figure 3:**
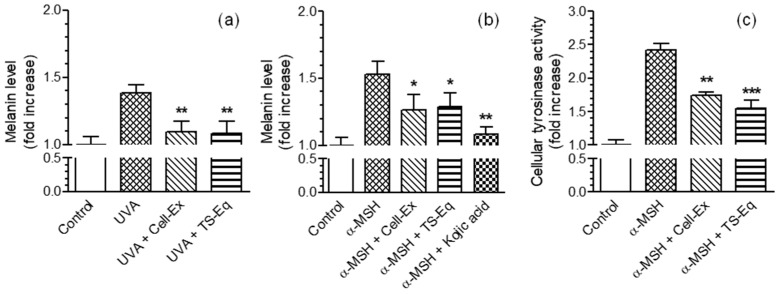
Effects of Cell-Ex and TS-Eq on melanin level and tyrosinase activity stimulated by UVA and α-MSH in B16-F10 cells. (**a**) Cells were treated with 10 µg/mL of Cell-Ex or TS-Eq and 20 J/cm^2^ of UVA. One hour after UVA exposure, the melanin levels were determined as reported in the Methods section. (**b**,**c**) Cells were treated with 10 µg/mL of Cell-Ex or TS-Eq and α-MSH (200 nM) for 72 h. At the end of treatment, melanin levels and tyrosinase activity were determined as reported in the Materials and Methods section. The melanin levels and tyrosinase activity values are expressed as fold increases with respect to untreated cells. The values are shown as the mean ± SD of three independent experiments (* *p* < 0.05, ** *p* < 0.01 and *** *p* < 0.001 vs. cells treated with t-BOOH or UVA via a one-way ANOVA with the Bonferroni post hoc test).

We therefore evaluated the ability of Cell-Ex and TS to inhibit the in vitro tyrosinase activity and decrease the melanin level in B16-F10 cells. Regarding the anti-tyrosinase activity, both Cell-Ex and TS were able to inhibit, in a concentration-dependent manner, the mushroom tyrosinase enzyme (Figure S5) with an IC50 of 11.77 µM for TS and 0.89 mg/mL for Cell-Ex. These results were in accordance with those reported by Dal Monte et al., who observed the anti-tyrosinase activity of a cell extract of *A. reptans* and of its main PGs teupolioside/isoteupolioside, of which the structures were very close to teucrioside [18]. After treating B16-F10 cells with 10 µg/mL of Cell-Ex and TS-Eq for 72 h, there was a significant reduction in melanin levels stimulated by either 20 J/cm^2^ of UVA (inhibition percentage, 76 vs. 79) or α-melanocytes-stimulating hormone, α-MSH (inhibition percentage, 51 vs. 45) (Figure 3a,b). Kojic acid, a well-known melanogenesis inhibitor, was used as a positive control at a dose of 500 µM in B16-F10 cells stimulated by α-MSH (Figure 2b). Ultrastructural analysis of BF16-F10 cells by transmission electron microscopy confirmed these findings (Figure S4). Cells treated with α-MSH displayed several stage IV mature melanosomes with melanin pigment granules scattered in the cytoplasm. In contrast, BF16-F10 cells treated with α-MSH and Cell-Ex exhibited only a few morphological structures associated with stage IV mature melanosomes. Then, the tyrosinase enzyme activity in BF16-F10 cells stimulated by α-MSH for 72 h in the presence of either 10 µg/mL of Cell-Ex or TS-Eq was evaluated. As shown in Figure 3c, the treatment of B16-F10 cells with both Cell-Ex and TS-Eq reduced the increase in tyrosinase activity evoked by α-MSH, with a maximum inhibition of 48% and 61%, respectively. Interestingly, we recorded that both Cell-Ex and TS-Eq had a similar pattern of maximum inhibition on the cellular tyrosinase activity at lower concentrations than those used in a cell-free system with mushroom tyrosinase enzyme. In this regard, the different levels of active concentrations suggest that Cell-Ex and TS-Eq could inhibit the tyrosinase enzyme and other kinases involved in its upstream activation of B16-F10 cells. A recent study with a similar experimental approach showed the ability of PG verbascoside to reduce the activation of the tyrosinase enzyme in B16-F10 cells by inhibiting adenyl cyclase and α-MSH signaling [17].

Taken together, these results show that Cell-Ex and TS-Eq can effectively prevent the production of melanin and the activity of the tyrosinase enzyme in BF16-F10 cells. Notably, the inhibitory effect was more pronounced in melanin levels stimulated by UVA compared to α-MSH, implying that the antioxidant properties of Cell-Ex and TS-Eq play a role in preventing tyrosinase activity under oxidative stress caused by UVA. However, further upstream and downstream mechanisms in melanogenesis pathways could contribute to Cell-Ex and its main PG TS anti-melanogenesis activity.

## 3. Materials and Methods

### 3.1. Chemicals

Azino-bis (3-ethylbenzothiazoline-6-sulfonic acid) diammonium salt (ABTS), 2′,7′-dichlorodihydrofluorescein diacetate (DCFH-DA), mushroom tyrosinase enzyme, α-Melanocyte-Stimulating Hormone (α-MSH), Levodopa (L-DOPA), L-Tyrosine, tert-butyl hydroperoxide solution (t-BOOH), 3-(4,5-dimethyl-2-thiazolyl)-2,5-diphenyl-2H-tetrazolium bromide (MTT) and phenylmethylsulfonyl fluoride were purchased from Sigma-Aldrich (Sigma-Aldrich, St. Louis, MO, USA). All chemicals used were of high-purity analytical grade.

### 3.2. Cell Culture and Stock Solutions

Murine melanoma B16F10 cells were obtained from Cell Bank Interlab Cell Line Collection (ICLC, IRCCS San Martino Policlinico Hospital, Genova, Italy) and cultured in Dulbecco’s modified Eagle medium supplemented with 10% fetal bovine serum, 2 mM glutamine, 50 U/mL penicillin and 50 μg/mL streptomycin at 37 °C in a humidified incubator with 5% CO_2_. Leaf-Ex and Cell-Ex stock solutions were prepared in dimethyl sulfoxide (DMSO) at 10 mg/mL. The stock solution of pure TS was prepared in 70% ethanol at 10 mg/mL. The stock solutions were diluted in a complete medium to get the requested concentrations of Leaf-Ex, Cell-Ex and TS in a maximum of 0.1% DMSO or ethanol.

### 3.3. Determination of Cell Viability

The cell viability was assessed by an MTT assay, as previously reported [19]. B16-F10 cells were seeded in a 96-well plate at 2 × 10^4^ cells/well and incubated for 24 h at 37 °C in 5% CO_2_. The cells were then treated with various concentrations of Leaf-Ex and Cell-Ex (1–100 µg/mL) for 24 and 72 h at 37 °C in 5% CO_2_. The treatment medium was then replaced with MTT in Hank’s Balanced Salt Solution (HBSS) (0.5 mg/mL) for 2 h at 37 °C in 5% CO_2_. After washing with HBSS, formazan crystals were dissolved in isopropanol. The formazan level was estimated (570 nm, reference filter 690 nm) using the multilabel plate reader VICTOR™ X3 (PerkinElmer, Waltham, MA, USA). Values are expressed as a percentage relative to untreated cells.

### 3.4. UVA Irradiation

The UVA irradiation was achieved with a bank of two Philips TLK 40W/10R fluorescence tubes (Sara s.r.l., Varese, Italy), emitting 350–400 nm energy, peaking at 365 nm, as previously reported. The fluorescence tubes were positioned to deliver 4095 mW/cm^2^ of UVA, as measured by a radiometer (Oriel Instruments, Stratford, CT, USA).

### 3.5. Determination of Antioxidant Activity

The antioxidant activity of Leaf-Ex, Cell-Ex and TS was evaluated in B16-F10 cells as previously described [20]. Briefly, B16-F10 cells were seeded in a 96-well plate at 3 × 10^4^ cells/well and incubated for 24 h at 37 °C in 5% CO_2_. The cells were then loaded with the fluorescent probe DCFH-DA (10 μg/mL) for 30 min at room temperature. At the end of incubation, the cells were treated with 10 µg/mL of Leaf-Ex, Cell-Ex and t-BOOH (100 μM) for 30 min. In parallel, another set of cells was exposed to 5 J/cm^2^ of UVA in the presence of Leaf-Ex, Cell-Ex (10 µg/mL), and TS-Eq. The ROS formation was determined at the end of the different treatment (excitation at 485 nm and emission at 535 nm) using a VICTOR™ X3 multilabel plate reader. The values of ROS formation are expressed as fold increases with respect to untreated cells.

### 3.6. Determination of the Melanin Levels

The melanin levels were determined using the previously described method [21]. Briefly, B16-F10 cells were seeded in culture dishes at 3 × 10^5^ cells/dish and incubated for 24 h at 37 °C in 5% CO_2_. The cells were treated with α-MSH (200 nM) and Cell-Ex (10 µg/mL) or TS-Eq for 72 h at 37 °C in 5% CO_2_. In parallel, another set of cells were exposed to 20 J/cm^2^ of UVA in the presence of Cell-Ex (10 µg/mL) or TS-Eq and further incubated for 1 h at 37 °C in 5% CO_2_. Kojic acid (700 µM) was used as a positive control. After washing twice with phosphate-buffered saline (PBS), the cells were centrifuged for 8 min at 10,000× *g* at 4 °C. Then, the supernatant was removed, and the pellet was dissolved in 1 mL of 1 N NaOH. To measure the melanin levels, 100 μL solution aliquots were placed in 96-well plates and the absorbance was measured at 450 nm using a VICTOR™ X3 multilabel plate reader. The values of the melanin levels are expressed as fold increases with respect to untreated cells.

### 3.7. Determination of Cellular Tyrosinase Activity

Cellular tyrosinase activity was determined using the previously described method [21]. Briefly, B16-F10 cells were seeded at a density of 5 × 10^4^ cells/well in 24-well plates and incubated for 24 h at 37 °C in 5% CO_2_. The cells were then treated with α-MSH (200 nM) and Cell-Ex (10 µg/mL) or TS-Eq for 72 h at 37 °C in 5% CO_2_. At the end of treatments, the cells were washed with ice-cold PBS and then lysed with 100 μL phosphate buffer (pH 6.8) containing 1% Triton X-100 and 0.1 mM phenylmethylsulfonyl fluoride. Then, lysate was centrifuged at 800 rpm for 5 min. The supernatant (100 μL) was added into 50 μL of L-DOPA (1 mM) and 50 μL of L-Tyrosine (2 mM), and the mixtures were placed in a 96-well plate. During the incubation at 37 °C, the absorbance was read at 490 nm every 30 min for 3 h using a VICTOR™ X3 multilabel plate reader. The values of tyrosinase activity are expressed as fold increases with respect to untreated cells.

### 3.8. Ultrastructural Analysis by Transmission Electron Microscopy (TEM)

B16-F10 cells treated for 72 h with α-MSH (200 nM) and Cell-Ex (10 µg/mL) were preliminarily fixed with 2.5% (*v*/*v*) glutaraldehyde in 0.1 M cacodylate buffer for 2 h and then fixed again with a solution of 1% (*w*/*v*) osmium tetroxide in 0.1 M cacodylate buffer. The cells were then subjected to a series of dehydration steps with different acetone solutions and then included in an epoxy resin. The embedded cells were cut into ultrathin slices, stained by uranyl acetate solution and lead citrate, and then observed by transmission electron microscope CM10 Philips (FEI Company, Eindhoven, The Netherlands) at an accelerating voltage of 80 kV. Images were recorded using a Megaview III digital camera (FEI Company, Eindhoven, The Netherlands). TEM analysis was performed in duplicate for each experimental point.

### 3.9. Determination of Mushroom Tyrosinase Activity

The activity of mushroom tyrosinase (EC 1.14.18.1) was determined according to the previously described method [22]. The percentage inhibition of enzyme activity was calculated using the following formula: %inhibition [1 − (Δ Abs/min sample/Δ Abs/min negative control) × 100], where the negative control was made with water instead of TS/Cell-Ex, and the positive control was performed with Kojic acid (KA), a well-known tyrosinase inhibitor.

### 3.10. Establishment of Suspension Cultures from Plant Leaves

Cell suspension cultures of *Teucrium chamaedrys* L. were obtained from friable callus cultures, which were established from fresh leaves, as previously described [9]. Suspension cultures were maintained on a rotary shaker (110 rpm) in a growth chamber at 22 °C under a 16/8 h photoperiod (white, fluorescent light at 50 µmol m^2^ s^−1^), and regularly sub-cultured every 2 weeks on a Murashige–Skoog (MS) medium supplemented with growth regulators. Elicitation was carried out by treating the cells with methyl jasmonate 0.5 mM at the seventh day from subculture, and exposing them to the elicitor for 24 h, as previously described [9]. The cells were then harvested by filtration, weighed, and immediately extracted.

### 3.11. Extract Preparation and HPLC-DAD Analysis

Extracts from cell cultures or leaves were prepared by homogenizing 1.0 g of fresh material with 5.0 mL of methanol in an Ultra-Turrax. The suspension was centrifuged at 1700 × *g* for 5 min, and the supernatant was collected. After three subsequent cycles, supernatants were combined, the solvent was evaporated under vacuum and the dried extract was then resuspended in DMSO to get a 0.5 mg/ mL concentration. A stock solution 0.1 mg/mL TS was prepared in methanol. PGs in both cell and leaf extracts were identified and quantified by HPLC-DAD using a Zorbax Eclipse Plus C18 reversed-phase column (100 mm × 3 mm I.D., 3.5 µm), under gradient conditions. The mobile phase consisted of A (0.1% formic acid in water) and B (0.1% formic acid in acetonitrile), and a gradient was applied according to Lin et al. [23]. The column oven temperature was set at 20 °C. The signal at 335 nm was used for quantitative purposes. Data were acquired and processed by the ChromNAV 2.0 HPLC Software (Jasco, Tokyo, Japan).

### 3.12. Statistical Analysis

Statistical analysis was done using PRISM 5 software (GraphPad Software, La Jolla, CA, USA). Student’s *t*-test and a one-way ANOVA with the Bonferroni post hoc test were chosen as the tests for data analysis.

## 4. Conclusions

Our results demonstrate for the first time the antioxidant and anti-melanogenic activity of a *T. chamaedrys* cell suspension extract and its main component PG TS in B16-F10 cells. Notably, Cell-Ex exhibits a biological effect profile, such as the inhibition of UVA-evoking an increase in ROS and melanin levels, like isolated TS, suggesting either its use as a raw material for the development of cosmetic ingredients or the use of *T. chamaedrys* cell suspension culture as a source of bioactive PG TS. In particular, these results provide evidence of the potential for using a biotechnological approach to produce an extract with added value for cosmeceutical use in protecting against photooxidative damage and hyperpigmentation. However, further in-depth studies are warranted to delineate the molecular mechanism of melanogenesis inhibition of Cell-Ex in primary human melanocytes or pigmented skin models.

Overall, these results show that TS has an interesting bifunctional activity relating to oxidative stress and tyrosinase activity, suggesting its potential uses in pharmaceutical and nutraceutical sectors, pure or botanical remedies beyond cosmeceutical applications. Therefore, TS could have potential therapeutic values in the treatment of a wide range of oxidative stress-mediated pathological conditions such as neurodegenerative, cardiovascular and inflammatory diseases, as well as chemopreventive activity for some types of tumors. Since tyrosinase is a rate-limiting enzyme in several undesirable melanogenesis processes, TS could also contribute to preventing fruit and vegetable browning, bacterial activity, cancer and Parkinson’s disease [24]. These potential therapeutic effects can prompt future research directions regarding the biological activities of TS.

## Data Availability

The data presented in this study are available on request from the corresponding author.

## Supplementary Materials

The following supporting information can be downloaded at: https://www.mdpi.com/article/10.3390/plants13060808/s1, Figure S1: Absorption spectrum of Cell-Ex; Figure S2: Absorption spectrum of Leaf-Ex; Figure S3: Absorption spectrum of TS; Figure S4: Ultrastructural analysis of B16-F10 cells by TEM; Figure S5: The effects of Cell-Ex and pure TS on mushroom tyrosinase activity.
