# Supplementary material for: Antioxidant and Anti-Melanogenesis Effects of Teucrium chamaedrys L. Cell Suspension Extract and Its Main Phenylethanoid Glycoside in B16-F10 Cells
Source: Plants (Basel). 2024 Mar 12;13(6):808. doi: 10.3390/plants13060808 (PMC10974463; doi:10.3390/plants13060808)
Supplement: Supplementary file 1 [file plants-13-00808-s001.zip › plants-2894275-supplementary.pdf]

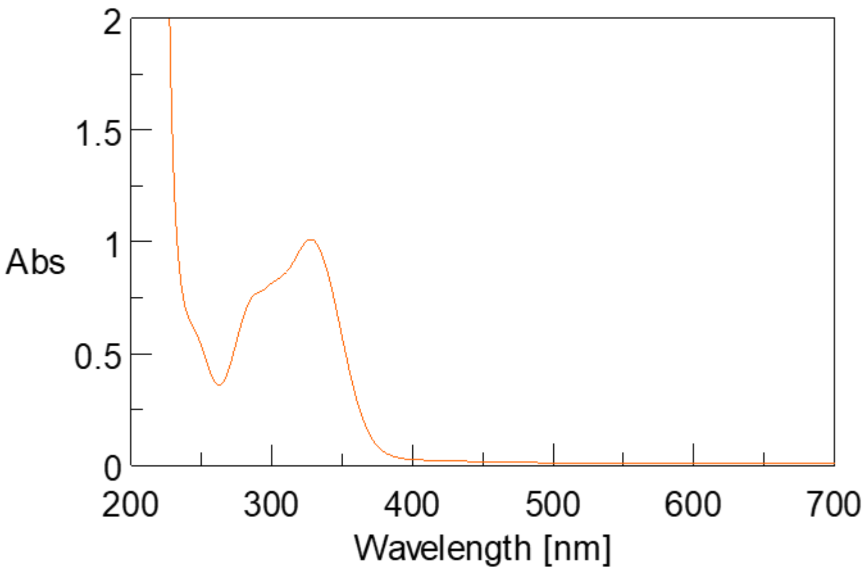

Figure S1. Absorption spectrum of Cell-Ex.

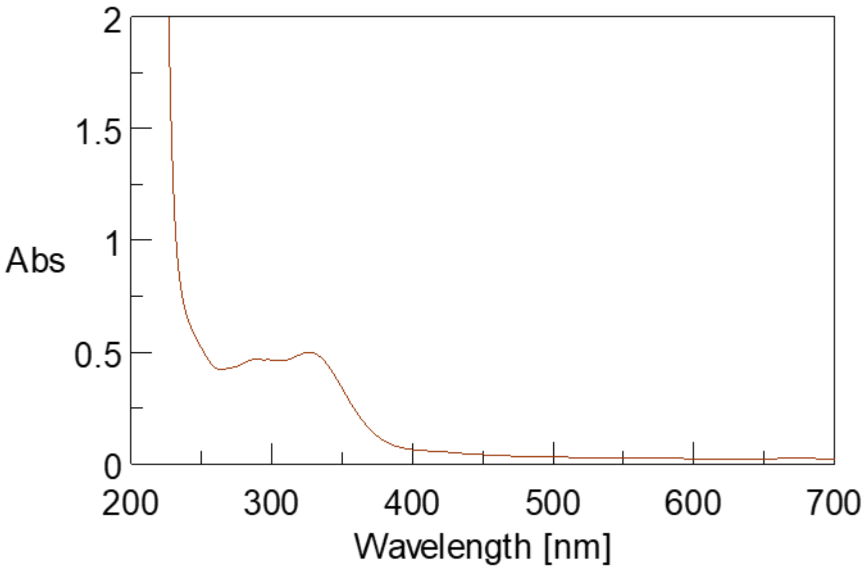

Figure S2. Absorption spectrum of Leaf-Ex.

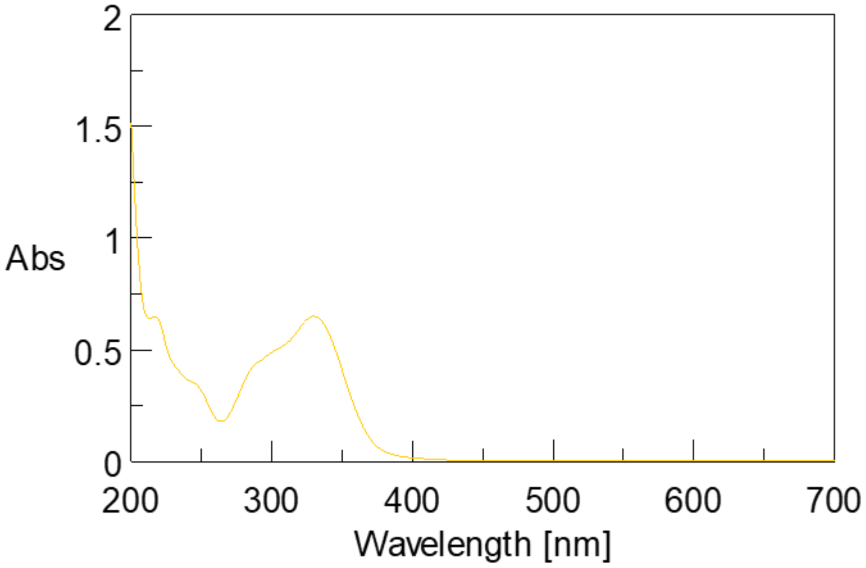

Figure S3. Absorption spectrum of TS.

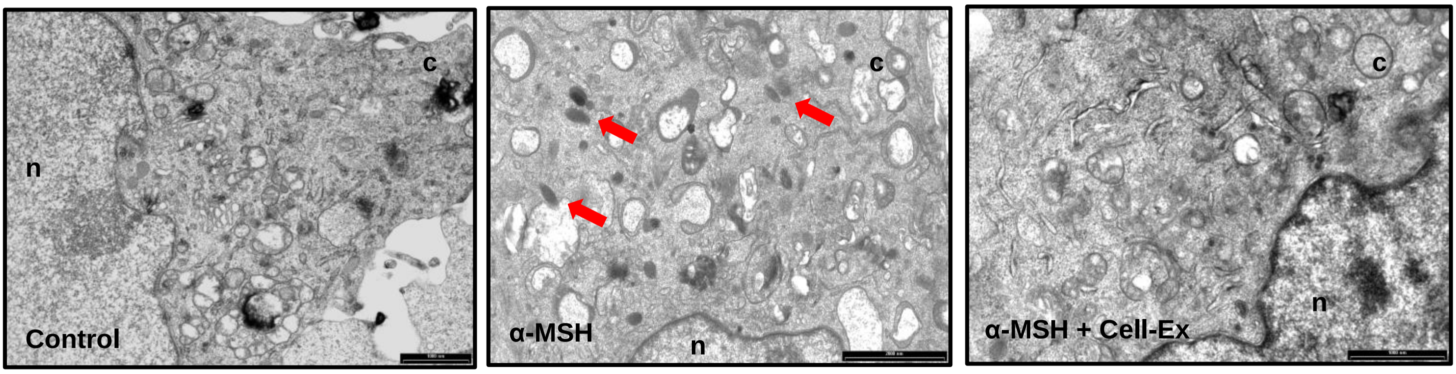

Figure S4. Ultrastructural analysis of B16-F10 cells by TEM (nucleus, n; cytoplasm, c). The red arrows show an example of mature melanosomes (stage IV) (bar: 2000 nm).

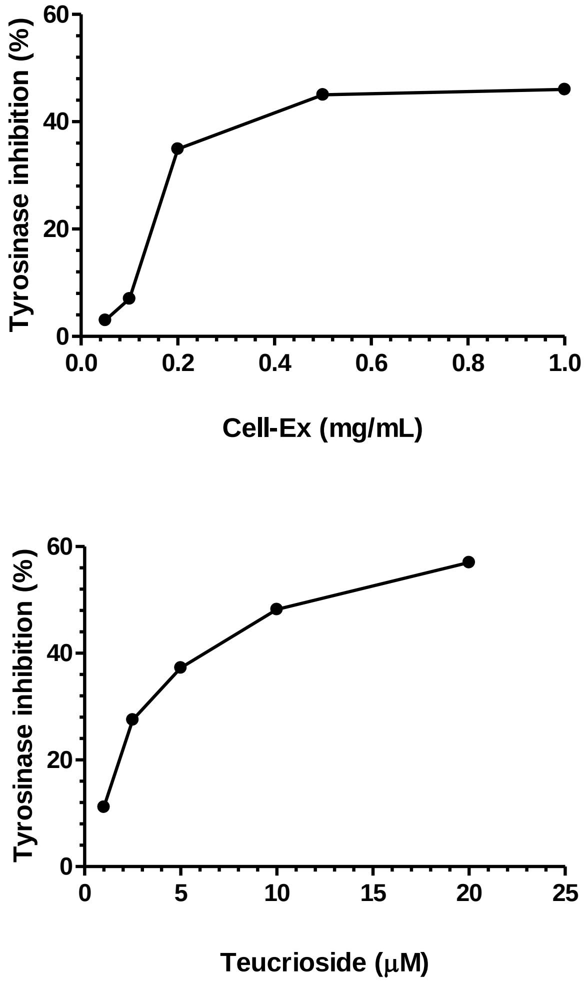

Figure S5. The effects of Cell-Ex and pure TS on mushroom tyrosinase activity.
